# Visual perception of highly memorable images is mediated by a distributed network of ventral visual regions that enable a late memorability response

**DOI:** 10.1371/journal.pbio.3002564

**Published:** 2024-04-01

**Authors:** Benjamin Lahner, Yalda Mohsenzadeh, Caitlin Mullin, Aude Oliva

**Affiliations:** 1 Computer Science and Artificial Intelligence Laboratory, Massachusetts Institute of Technology, Cambridge, Massachusetts, United States of America; 2 The Brain and Mind Institute, The University of Western Ontario, London, Canada; 3 Department of Computer Science, The University of Western Ontario, London, Canada; 4 Vector Institute for Artificial Intelligence, Toronto, Ontario, Canada; 5 Vision: Science to Application (VISTA), York University, Toronto, Ontario, Canada; Vanderbilt University, UNITED STATES

## Abstract

Behavioral and neuroscience studies in humans and primates have shown that memorability is an intrinsic property of an image that predicts its strength of encoding into and retrieval from memory. While previous work has independently probed when or where this memorability effect may occur in the human brain, a description of its spatiotemporal dynamics is missing. Here, we used representational similarity analysis (RSA) to combine functional magnetic resonance imaging (fMRI) with source-estimated magnetoencephalography (MEG) to simultaneously measure when and where the human cortex is sensitive to differences in image memorability. Results reveal that visual perception of High Memorable images, compared to Low Memorable images, recruits a set of regions of interest (ROIs) distributed throughout the ventral visual cortex: a late memorability response (from around 300 ms) in early visual cortex (EVC), inferior temporal cortex, lateral occipital cortex, fusiform gyrus, and banks of the superior temporal sulcus. Image memorability magnitude results are represented after high-level feature processing in visual regions and reflected in classical memory regions in the medial temporal lobe (MTL). Our results present, to our knowledge, the first unified spatiotemporal account of visual memorability effect across the human cortex, further supporting the levels-of-processing theory of perception and memory.

## Abstract 2

How does the processing of images with high and low memorability differ in the brain? This study combines functional magnetic resonance imaging and magnetoencephalography to show that only images with high memorability scores undergo extended processing late in time throughout the ventral visual stream, including early visual cortex.

## 1. Introduction

Although visual memorability feels at first a subjective and hard to quantify property of an image, many behavioral, neuroscience, and computational works have shown that it is not an inexplicable phenomenon—we can calculate an image’s memorability score as its probability of being correctly remembered at a later viewing. Visual memorability has been shown to be intrinsic to the stimulus and reproducible across individuals. This means that despite varied experiences, individuals tend to remember and forget the same images [1–7]. This suggests images with a high memorability score are better encoded into and retrieved from memory not due to individual preferences, but due to perceptual properties of the stimuli [2,8–10].

Measuring these perceptual differences between images with high and low memorability scores can support or discourage theories relating perception and memory processes. Differences arising in the first few hundred milliseconds of perception within perceptual regions may better align with the levels-of-processing theory of memory, which posits that memory of a stimulus is a result of deeply processing the stimulus during perception [11,12]. Differences arising in non-perceptual regions temporally distinct from perception may better align with the multi-store models of memory, which states that stimulus processing and memory storage are separate [13]. A spatiotemporal account of the memorability effect would allow for controlled examination of this crucial link between perception and memory.

The current spatiotemporal account of memorability sensitivity is coarse and may be incomplete, relying on results pieced together from independent unimodal experiments with different subjects, stimulus sets, and tasks. Previous work spatially localizes memorability sensitivity to ventral and medial temporal lobe (MTL) regions [8–10] and temporally localizes it to a middle stage of visual perception, at around 150 ms poststimulus onset [14]. When specific regions of interest (ROIs) become sensitive to memorability relative to both stimulus onset and other perceptual processes remains unknown.

Here, we describe the dynamics of image memorability in humans in high spatiotemporal resolution by combining 2 neuroimaging techniques, magnetoencephalography (MEG) and functional magnetic resonance imaging (fMRI). This method is known as MEG-fMRI fusion [15–21]. We isolate the effect of image memorability using semantically matched High Memorable and Low Memorable stimulus sets controlled for low-level features (see S1 Table). In doing so, we provide a unified account of the spatiotemporal dynamics of the memorability effect throughout the whole brain and identify which brain regions are sensitive to memorability and when.

To anticipate our results, we find that images with high memorability scores recruit a distributed network of ventral visual regions for temporally extended processing late in time, beginning at approximately 250 ms poststimulus onset. We identify 5 ROIs, inferior temporal cortex, fusiform gyrus, lateral occipital cortex, banks superior temporal sulcus (banks of the STS), and, notably, early visual cortex (EVC), as showing significant sensitivity to image memorability. We provide evidence that image memorability overlaps with both visual perceptual and memory regions by measuring how well a representation derived from human-based memorability score magnitudes matches neural representations in classical visual and memory regions. We find the memorability magnitude representation arises after high-level image feature processing but still during the perceptual process and memory regions in the MTL (hippocampus, amygdala, and parahippocampal gyrus) significantly represent memorability judgments. Together, our results provide a detailed spatiotemporal account of the memorability effect at the voxel and region-level and support the levels-of-processing theory linking perception with memory.

## 2. Results

### 2.1. MEG-fMRI fusion maps of the time course of the memorability effect in the brain

**Fig 1:**
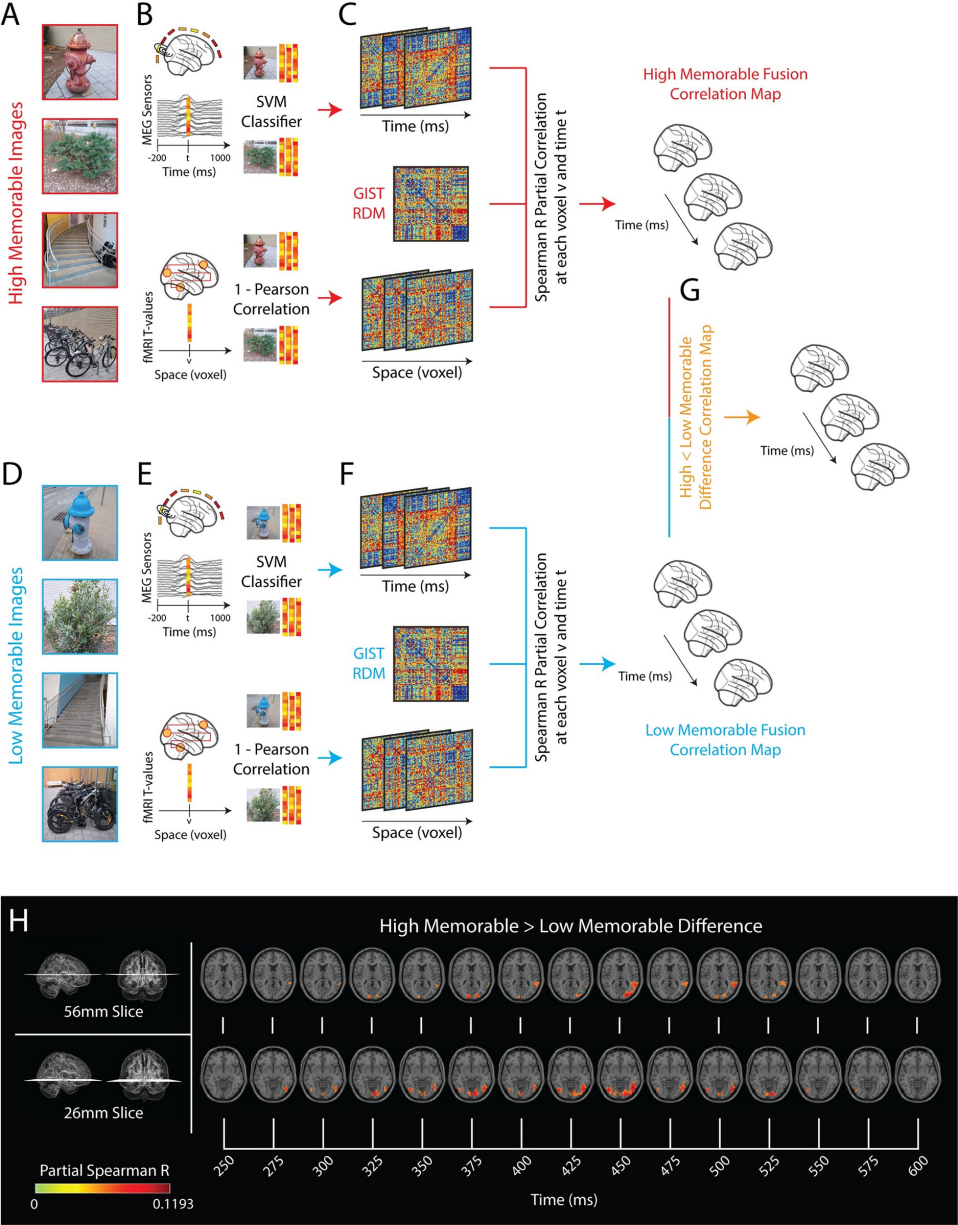
Whole-brain MEG-fMRI fusion methodology and results. ** (A, D) Stimuli examples:** Illustrative examples of experimental stimuli and their High and Low Memorable pairs of the same semantic concept. For example, the bottom images in panels **(A)** and **(D)** show a High Memorable image of “a bike rack outside of a building” and a Low Memorable image of “a bike rack outside of a building,” respectively. The real experimental stimuli are not shown due to copyright protection. The analysis steps **(BC)** and **(EF)** were separately performed on the 78 High Memorable conditions and the 78 Low Memorable conditions, respectively, and are otherwise identical. **(B, E) Computing pairwise dissimilarities:** For the MEG experiment, at each time point *t*, an RDM was computed where the (i,j) indices correspond to a dissimilarity measure (SVM classification accuracy) between condition *i* and condition *j*. For the fMRI experiment, at each voxel *v*, an RDM was computed where the (i,j) indices correspond to a dissimilarity measure (1-Pearson’s R) between the activation patterns within a spherical searchlight (radius of 4 voxels). **(C, F) MEG-fMRI fusion correlation map:** The partial correlation (Spearman’s R) between the MEG RDMs at each time point *t* and the fMRI RDMs at each voxel *v* while regressing out a GIST image feature RDM results in a time series of correlations across the whole-brain while controlling for possible confounding image features. **(G) High Memorable and Low Memorable correlation map difference:** The Low Memorable correlation maps were subtracted from the High Memorable correlation maps to create a new High > Low Difference correlation map over the whole-brain and across time. **(H) High > Low Memorable fusion results:** Axial slices of the brain at 56 mm and 26 mm between 250 ms and 600 ms poststimulus onset depict significant differences in correlation between the High Memorable and Low Memorable correlation maps (*n* = 15, cluster-definition threshold *P* < 0.001, significance threshold (alpha) *P* < 0.01, permutations = 1,000). fMRI, functional magnetic resonance imaging; MEG, magnetoencephalography; RDM, representational dissimilarity matrix; SVM, support vector machine.

The effect of image memorability has typically been studied in either time or space, but not both. Here, we document when and where the effect of image memorability propagates throughout the whole-brain with MEG-fMRI fusion [15,16,19,22], a technique that enables comparison of MEG and fMRI data by mapping them into a common representational space through the calculation of representational dissimilarity matrices (RDMs) [23] (Fig 1B and 1E). By correlating a voxel’s fMRI RDM with each time point’s MEG RDM, we estimate that voxel’s contribution to the MEG signal over time. Repeated for every voxel in the brain, we achieve a matrix of correlations describing visual processing over the whole-brain and time (Fig 1C and 1F).

We compute an MEG-fMRI fusion correlation series on the 78 High Memorable conditions (Fig 1A–1C) and 78 Low Memorable conditions (Fig 1D–1F) separately. Both MEG-fMRI fusion correlation series show significant clusters of correlations onset in the EVC around 80 ms and propagate throughout the visual cortex, as expected from previous works [15,21] (see S1 Movie).

We then subtract the Low Memorable fusion correlation series from the High Memorable fusion correlation series to obtain a spatiotemporal view of sensitivity to image memorability (Fig 1G). Since the High and Low Memorable stimuli sets are controlled for high- and low-level perceptual features (see S1 Table), any effect that remains after the High > Low Memorable difference contrast is attributable to the difference in memorability scores. Furthermore, this difference contrast is not biased by hand-selected memorability features in order to provide a general description of memorability sensitivity throughout the whole brain.

Fig 1H shows snapshots of the whole-brain High Memorable > Low Memorable difference fusion (cluster statistics) over the time points 250 to 600 ms in 2 select axial slices highlighting significant differences in correlations distributed throughout the visual cortex. Significant differences in correlations first arise in the posterior temporal lobe at around 275 ms followed by responses in the posterior occipital lobe and lateral occipital lobe at 300 ms. Significant differences in correlations cease by 600 ms (see S1 Movie for a link to the High Memorable, Low Memorable, and High > Low Memorable difference whole-brain MEG-fMRI fusion movies side-by-side).

Overall, results from this spatially and temporally unconstrained fusion analysis provide evidence that sensitivity to image memorability is distributed throughout visual cortex late in time. This whole-brain MEG-fMRI fusion analysis complements previous MEG-only work showing sensitivities to image memorability late in time [14] and fMRI-only work showing sensitivity to image memorability near high-level ventral visual stream and posterior temporal lobe (but not in extra-visual regions associated with memory) [8,9]. In contrast to these previous works, we identify voxels sensitive to image memorability in the posterior occipital lobe, a region typically associated with low-level image feature processing. The late and distributed responses warrant region-specific analyses to further quantify onset times and build a clearer picture of the effect of image memorability on visual perception.

### 2.2. ROI-based MEG-fMRI fusion shows late onset of image memorability sensitivity

We extend the MEG-fMRI fusion analysis to individual ROIs defined for both the fMRI and MEG data to identify which regions are sensitive to image memorability and when they show this sensitivity. We identify 6 non-overlapping ROIs from the Desikan–Killiany atlas [24] to spatially constrain both the MEG and fMRI data. Specifically, we use the ROIs EVC (combination of lingual gyrus, cuneus cortex, and pericalcarine cortex regions), lateral occipital cortex, inferior temporal cortex, fusiform gyrus, middle temporal gyrus, and banks of the STS for their non-overlapping but abutting coverage in or near the ventral visual stream that we beforehand might expect to be sensitive to image memorability [8–10,14]. These ROIs span typical visual regions (EVC, lateral occipital cortex, inferior temporal cortex, fusiform gyrus) [25–31] and nearby multimodal integration regions (middle temporal gyrus and banks of the STS) [32–36] for broad functional coverage of visual perception.

**Fig 2:**
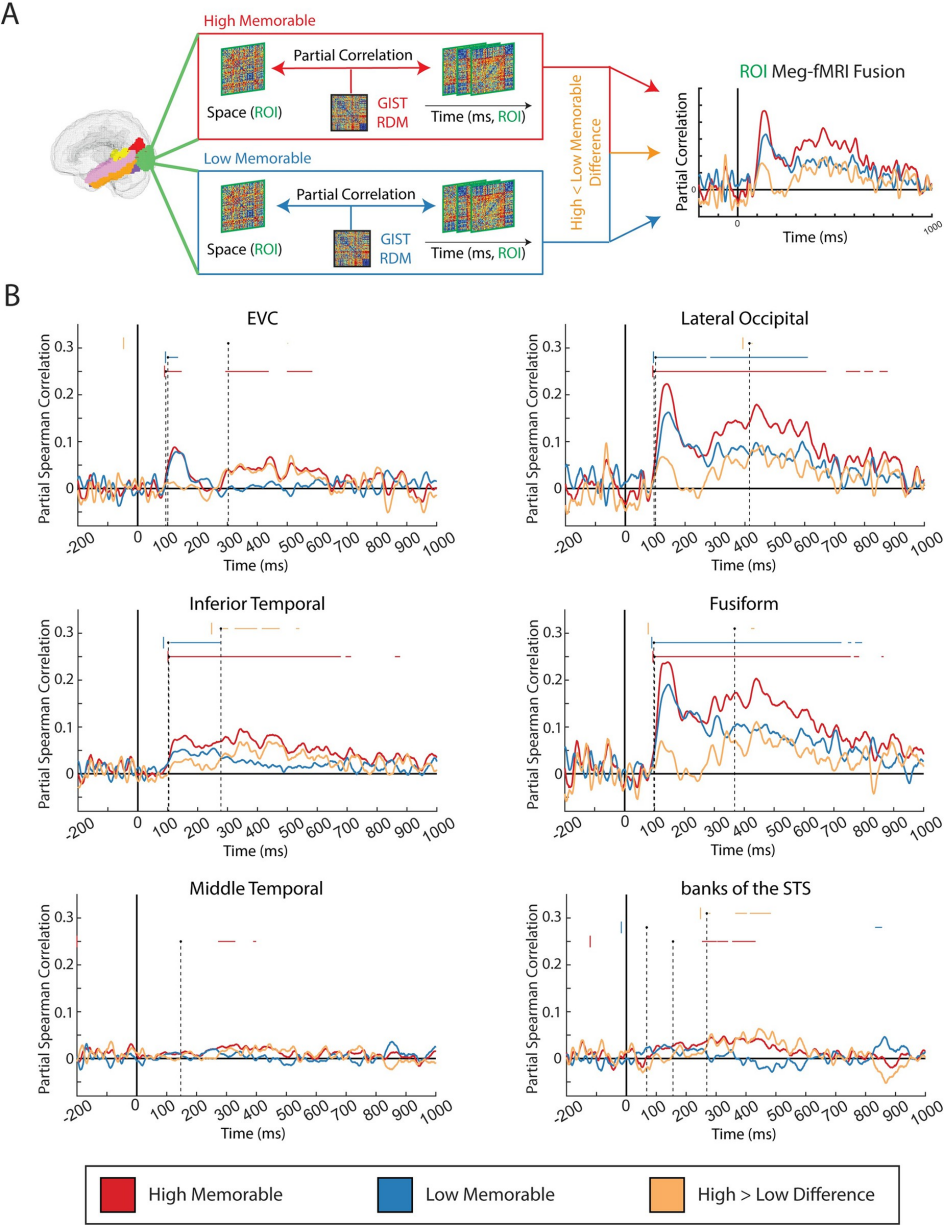
Source-estimated MEG-fMRI ROI fusion correlation time series. **(A) ROI MEG-fMRI fusion methods:** For each of the 6 ROIs (in this example, the green ROI is lateral occipital), an fMRI RDM and a source-estimated MEG RDM time series are partially correlated (regressing out the GIST image features) for the High Memorable (red) and Low Memorable (blue) images, separately. The resulting Low Memorable fusion correlation curves are subtracted from the High Memorable fusion correlation curves to compute the High > Low Memorable Difference curve (orange). **(B) Source-estimated MEG-fMRI ROI fusion results:** The 15-subject grand average correlation curves for the High Memorable, Low Memorable, and High > Low Memorable Difference conditions are plotted for 6 ROIs in or abutting the ventral visual stream. The corresponding color-coded horizontal bars above the curves represent the significant time points at which all 15 jackknifed subsamples passed the jackknife-based baseline criteria. The black dots depict the average onset within each High, Low, and High > Low condition, and the color-coded vertical bar depicts the onset’s 95% confidence interval (one-sample *t* test, right-sided, variance corrected for the subsamples). The dotted black vertical lines connect each conditions’ average onset to the x-axis for easier viewing of the onset times. fMRI, functional magnetic resonance imaging; MEG, magnetoencephalography; RDM, representational dissimilarity matrix; ROI, region of interest.

The ROI-based MEG-fMRI fusion method (see section 4.8.2) correlates a source-estimated MEG time series of RDMs (see section 4.6.3) with an fMRI RDM (see section 4.7.3) for a given ROI (Fig 2A, example green ROI is lateral occipital cortex) to obtain a ROI-specific correlation time series for each subject. This procedure is done separately for the High Memorable (red) and Low Memorable conditions (blue) (Fig 2A). Subtracting the Low Memorable correlation time series from the High Memorable correlation time series results in the High > Low Memorable Difference correlation time series (orange) (Fig 2A). We estimate onset latencies for the High, Low, and High > Low Difference conditions within each ROI using a jackknife-based baseline technique [37–40]. In detail, the jackknife-based baseline technique computes leave-one-out subsamples and, for each subsample, considers a time point as significant if: (1) the value at that time point exceeds 2 times the baseline standard deviation; and (2) the average value over 10 consecutive 50 ms windows also exceeds 2 times the baseline standard deviation. The first time point that satisfies both criteria is marked as the onset for that subsample (see Methods section 4.8.2 for more details). Fig 2B shows the grand-average correlation time series curves, the average onset for each condition (the average of all subsample onsets), and the significant time points for the High, Low, and High > Low difference condition for each ROI (time points where all 15 jackknife subsamples met the 2 criteria).

Fig 2B shows the presence of significant time points in the High > Low Difference correlations for EVC, lateral occipital cortex, inferior temporal cortex, fusiform gyrus, and banks of the STS with an average onset of 303, 415, 278, 368, and 269 ms poststimulus onset, respectively (see S2 Table for results for all conditions). This result shows that perception of High Memorable images differs from perception of Low Memorable images in a spatially distributed set of regions late in time.

### 2.3. Memorability score magnitude is represented after high-level perception

**Fig 3:**
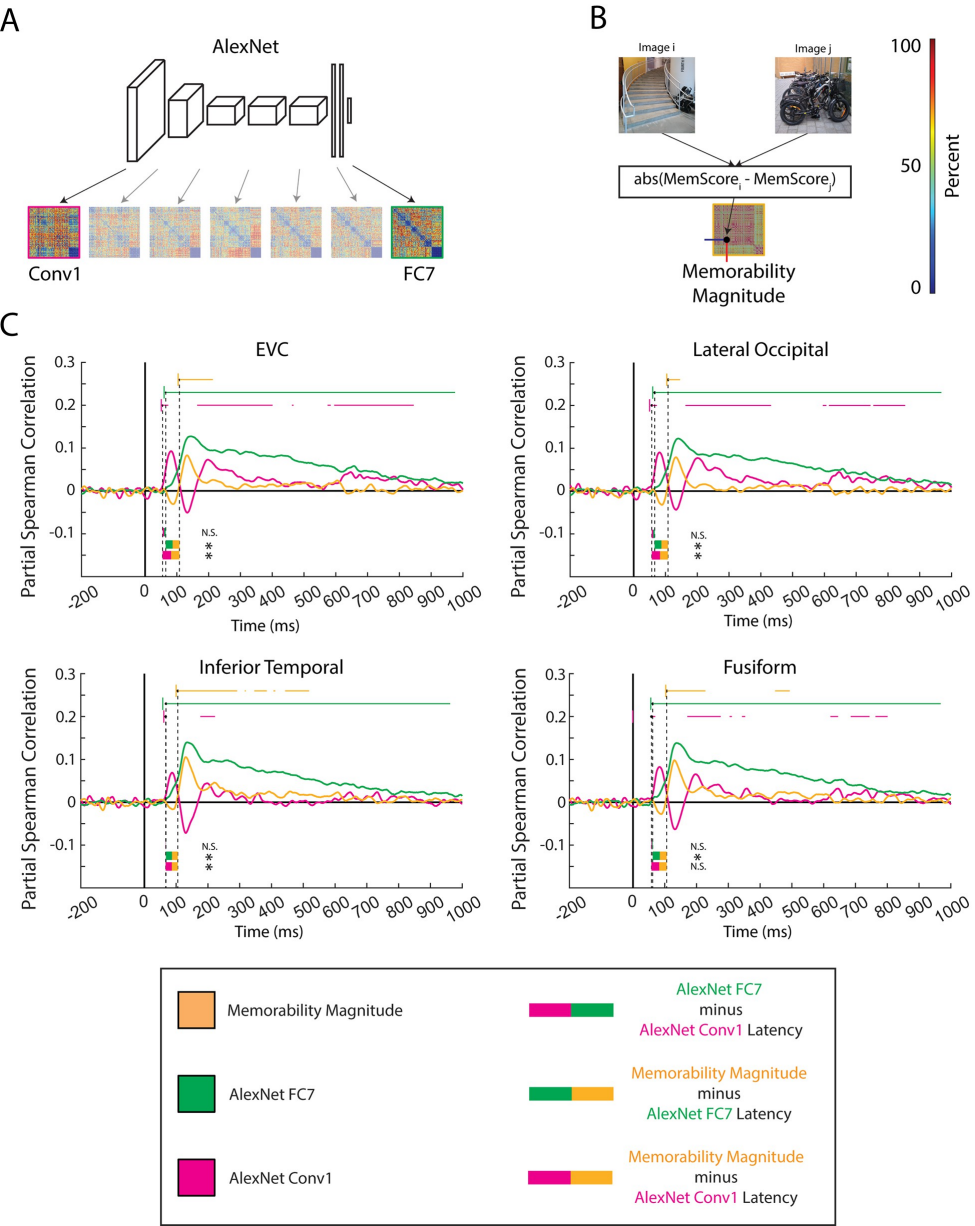
Model and source-estimated MEG RSA. **(A) Creation of AlexNet layer RDMs:** Features for each of the 156 stimuli were extracted after the first convolution (Conv1, pink) and after the penultimate fully connected layer (FC7, green) in an AlexNet model pretrained on ImageNet. Dissimilarity between each pair of images was measured as 1 –Pearson correlational similarity between the flattened image features and entered into the RDM. **(B) Creation of memorability behavior RDM:** Each value in the *(i*,*j)* index of the memorability behavior RDM (orange) is the absolute value of the image *i’*s memorability score minus image *j*’s memorability score. **(C) Correlation of model RDMs with MEG RMDs:** The three 156 × 156 model RDMs (AlexNet Conv1, AlexNet FC7, and memorability behavior) were partially correlated (spearman; Conv1 partialled out FC7, FC7 partialled out Conv1, and memorability behavior partialled out GIST) with the source-estimated MEG time series for each of the 4 typical visual regions. The color-coded horizontal bars above the curves represent the significant time points at which all 15 jackknifed subsamples passed the jackknife-based baseline criteria. The average onset time and 95% confidence interval for each curve are denoted by black dots with a dotted black line extending down to the x-axis (for easier visualization) and a short color-coded vertical bar, respectively. The onset times were computed using a jackknife approach. The latencies between condition onsets are denoted by dual color-coded bars below the curves. The latencies were computed using a two-sided *t* test and corrected for multiple comparisons using the Bonferroni–Holm procedure (q < 0.05, *n* = 12 comparisons). An asterisk to the right of the latency bar denotes significance, and N.S. denotes “Not Significant.” MEG, magnetoencephalography; RDM, representational dissimilarity matrix; RSA, representational similarity analysis.

In order to pinpoint when image memorability is represented with respect to the perceptual time course, we use representational similarity analysis (RSA) [23,41] to correlate low-level, high-level, and memorability behavior model RDMs with the source-estimated MEG RDM time series of classical visual processing regions [15,42–45]. To approximate the low-level and high-level image representations of the human visual system, RDMs are computed by extracting the features after the first convolutional layer (here referred to as Conv1) and penultimate fully connected layer (here referred to as FC7) from an AlexNet deep convolutional neural network (DNN) trained on image classification [46] (Fig 3A). To capture the human brain’s behavioral readout of image memorability, a model RDM is computed using the magnitude of difference in memorability scores between each pair of stimuli (Fig 3B).

Correlating a model RDM with a region’s source-estimated MEG RDM time series measures how strongly the ROI shares the model’s representation over time, indicative of the ROI’s underlying neural processes [23,41,42]. Crucially, the model and source-estimated MEG RDMs use all 156 image conditions to capture how images across High and Low Memorable conditions are represented. Comparing differences in onset latencies between each of these 3 model representations then allows us to establish a temporal ordering of these neural processes. We restrict this analysis to classical visual regions (EVC, lateral occipital cortex, fusiform gyrus, and inferior temporal cortex) for fair comparison with a vision-based model. We compute the conditions’ onsets (as opposed to peaks) to measure the earliest time the conditions are represented in the brain.

We find that the onset of image memorability processing (orange bar) is later than low-level (pink bar) and/or high-level (green bar) visual processing in all 4 visual regions by an average of 47 milliseconds and 42 milliseconds, respectively (Fig 3C). EVC, lateral occipital cortex, and inferior temporal cortex show the processing of memorability magnitudes occurs significantly later than both low-level and high-level processing, suggesting that the processing of image memorability is temporally distinct, and later than, both low-level and high-level perceptual processing (two-sided *t* test, Bonferroni–Holm correction for 12 comparisons, q < 0.05). In fusiform gyrus, the processing of memorability magnitudes occurs later than the high-level processing. Latency calculations involving the low-level features, especially latency differences between low-level and high-level features, may not have become significant due to larger variability in the low-level features (see S3 Table for all onset and latency values).

Together, this analysis shows that the image memorability representation that reflects behavioral recognition performance arises shortly after high-level visual processing (approximately 42 ms after) but still well within the time period of perceptual processing (approximately 107 ms poststimulus onset). Thus, the behavioral readout of image memorability is a late-stage representation but is still reflected during perception.

### 2.4. Memorability score magnitude is reflected in classical memory regions

To determine the extent that classical memory regions also represent image memorability, we create RDMs for the hippocampus, parahippocampal gyrus, amygdala, and left prefrontal cortex (LPFC) and correlate them with the memorability score magnitude RDM. The hippocampus, parahippocampal gyrus, and amygdala compose part of the MTL and are often implicated in encoding, retrieving, and/or mediating memory [47–52]. LPFC has been consistently observed to positively respond in subsequent memory literature [53] and used in prior memorability work to dissociate image memorability from individual memory [8].

**Fig 4:**
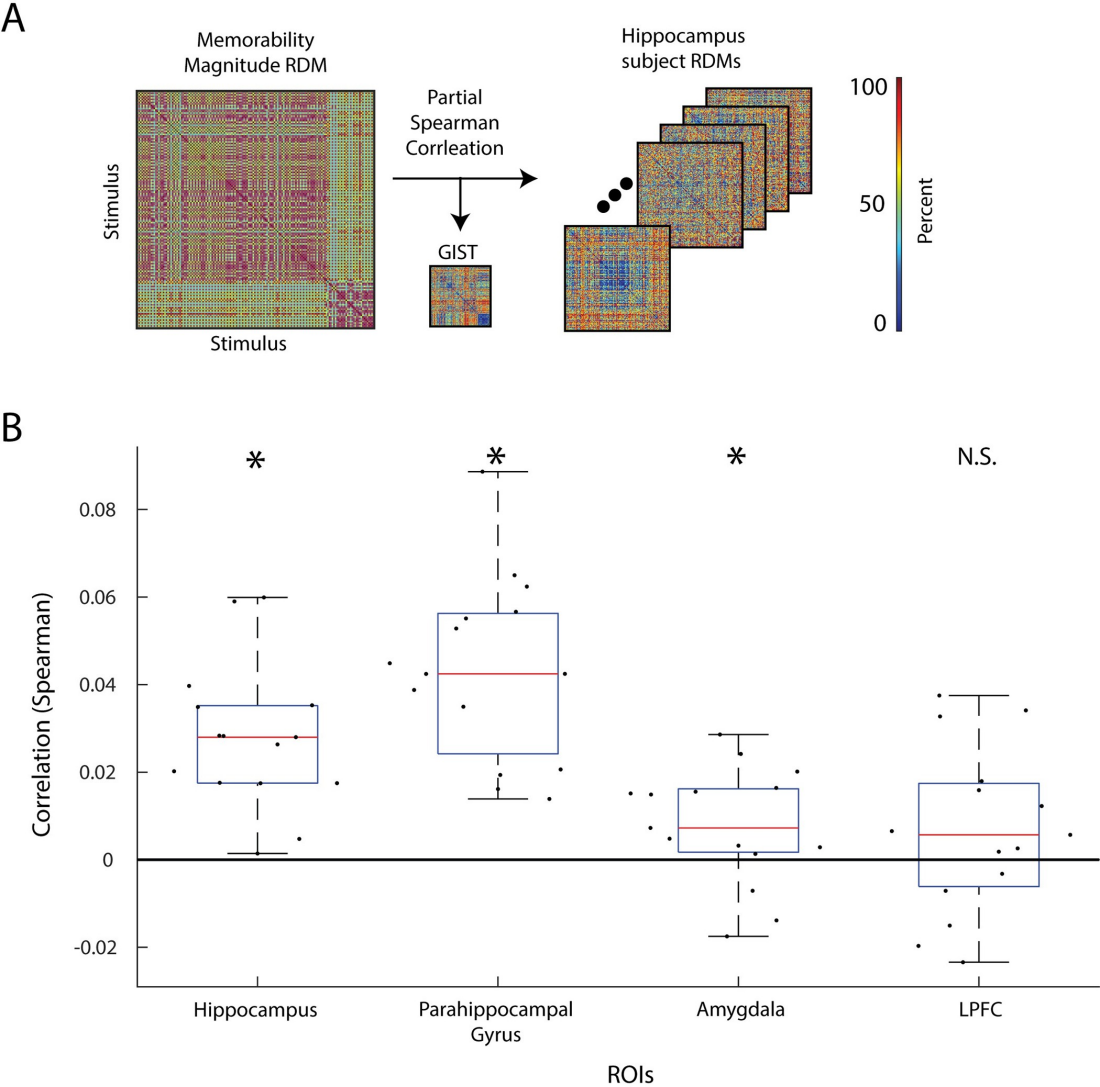
Correlation of classical memory fMRI RDMs with memorability magnitude RDM. **(A) Correlation analysis:** The 156 × 156 memorability magnitude RDM is partially correlated (spearman, partialling out GIST features) with each of the 15 subject’s fMRI ROI RDM (in this example, the hippocampus ROI). **(B) Memorability representation in classical memory regions:** For each of the 4 memory regions, the partial correlation (Spearman, GIST features partialled out) between the 15 subjects’ fMRI RDM and the memorability magnitude RDM was computed (black dots). Statistical significance was computed using a right sided *t* test against a null correlation of 0 (q < 0.05, Bonferroni–Holm correction, *n* = 4 comparisons). The boxplot shows the median correlation (red line), 25th and 75th percentile (blue box), and the most extreme non-outlier points (black whiskers). An asterisk above the boxplot denotes significance, and N.S. denotes “Not Significant.” fMRI, functional magnetic resonance imaging; LPFC, left prefrontal cortex; RDM, representational dissimilarity matrix; ROI, region of interest.

The memorability magnitude RDM (Fig 4A), also used in the analysis in section 2.3, summarizes the relationship between each pair of images by their difference in ability to be correctly remembered by human observers.

Similar to [8], we find that hippocampus (*p* = 2.1e-5), parahippocampal gyrus (*p* = 2.4e-6), and amygdala (*p* = 0.0427) significantly correlate with the memorability magnitude RDM, while LPFC (*p* = 0.1004) does not (right-sided *t* test, Bonferroni–Holm correction for *n* = 4 comparisons, q < 0.05; see S4 Table for equivalent nonparametric tests). This result shows the behavioral representation of image memorability is represented in not only visual regions (see section 2.3) but also memory regions.

## 3. Discussion

### 3.1. Summary of results

This study combines high spatial resolution fMRI with high temporal resolution MEG to provide a unified account of the when and where of the memorability effect throughout the whole brain. Spatiotemporal analyses reveal that sensitivity to image memorability is more distributed than previously thought. We find sensitivity in not only the high-level visual regions of inferior temporal cortex, fusiform gyrus, and lateral occipital cortex, but also the banks of the STS and EVC. This sensitivity occurs late in visual processing, at approximately 303, 415, 278, 368, and 269 ms for EVC, lateral occipital cortex, inferior temporal cortex, fusiform gyrus, and banks of the STS, respectively. We then analyze the extent classic visual regions and classic memory regions represent a specific model of image memorability based on humans’ image recognition performance. The visual brain represents memorability score magnitudes significantly after high-level features with its first representations emerging approximately 107 ms poststimulus onset, suggesting that the memorability effect arises late in, but during, perception. Additionally, classical memory regions of hippocampus, parahippocampal gyrus, and amygdala reflect representations of memorability score magnitudes, further showing how visual perception and memory are intertwined.

### 3.2. EVC shows sensitivity to image memorability

Both our whole-brain MEG-fMRI fusion (section 2.1) and ROI-based MEG-fMRI fusion (section 2.2) analyses exhibit strong agreement that EVC is sensitive to differences in image memorability late in time (around 303 ms poststimulus onset). This result is in contrast with previous work that identifies image memorability as a high-level process different from low-level vision and did not find significant involvement of EVC [8,9,14]. Why does this set of MEG-fMRI fusion analyses find significant sensitivity in EVC?

We first note that this study’s fMRI-only univariate and multivariate analyses (see S2 Fig and S3 Fig), closely resembling the analyses of Bainbridge and colleagues (2017) and Bainbridge and Rissman (2018), also do not appear to show sensitivity in EVC. However, these multivariate analyses show a lack of statistically significant correlation between an EVC voxel’s representational geometry and a specific memorability-related representational geometry. Such an analysis is not sufficient to conclude that EVC is not sensitive to any aspects of image memorability.

The MEG-fMRI fusion analysis, on the other hand, can be viewed as testing 1,201 representational geometries (one for each MEG time point) that do not necessarily reflect high-level theories of memorability, but rather representations defined by the MEG signal at a time point. Thus, it is likely that EVC is sensitive to image memorability, but its representational geometry corresponds to a more intermediate level of processing best brought out by the MEG time points’ representational geometries, not the memorability theories previously tested.

A second (not mutually exclusive) possible explanation as to why we see significant late correlational differences in EVC may be due to the temporal limitations of fMRI. The temporally coarse and sluggish BOLD response captured at each voxel in EVC, and subsequently its RDM, is likely dominated by a strong representational geometry corresponding to its initial low-level visual processing early in time. If the signal of EVC’s sensitivity to differences in image memorability is weaker than the signal of EVC’s initial low-level visual processing, the memorability effect’s representational contribution to the overall EVC RDM would be present but relatively weak. MEG’s temporally resolved representational geometries, as utilized in MEG-fMRI fusion analyses, may be able to highlight EVC’s memorability representation.

### 3.3. Spatiotemporal results support the levels-of-processing theory of memory

Our results support the levels-of-processing theory of memory, which posits that a stimulus’s better encoding into and retrieval from memory is a consequence of deeper levels of perceptual processing [11,12]. Results show the memorability effect is distributed across regions around the occipital cortex often implicated in perception (sections 2.1, 2.2, and 2.4). The memorability score magnitude RDM is first reflected in visual ROIs after high-level feature processing but still during perception (approximately 107 ms after stimulus onset) (section 2.3), and significant High Memorable and Low Memorable differences are observed at even later time points (approximately 300 ms depending on the ROI) (section 2.2). Finally, we show the memorability score magnitude RDM is significantly represented in both classical visual regions (section 2.3) and classical memory regions in the MTL (but not LPFC) (section 2.4). Together, we show that the memorability effect is observed in both visual and memory regions and characterized by spatially distributed and temporally extended perceptual processing strongly suggestive of increased processing depth [11,54].

Marrying our results with previous literature, we propose that both High and Low Memorable images are similarly processed in an initial feedforward pass through the visual perceptual hierarchy [55,56] to extract their low-level (e.g., spatial frequency, color, contrast) and high-level (e.g., shapes, concepts) properties. The high-level visual regions at the end stages of the initial visual perception, specifically the inferior temporal cortex (Fig 2B, middle left panel) and banks of the STS (Fig 2B, lower right panel) seen here at around 278 ms and 269 ms, respectively, may preferentially process High Memorable images due to their relatively more conceptually [27,57] and socially [33,34] useful information content [58].

Banks of the STS, with its strong feedback connections to EVC [59,60], may then project the signal back to EVC at approximately 303 ms (Fig 2B, top left). EVC may serve to extract additional fine details of High Memorable images for extended processing and eventual encoding to or retrieval from memory, in line with EVC’s known involvement in high-order perception [30,31,59,61,62], retention of fine details in memory [63–65], and our results showing temporally distinct onsets between low-level, high-level, and memorability behavior representations (see section 2.3). Thus, we hypothesize that High Memorable images are subject to deeper levels of processing in order to extract additional relevant details in line with levels-of-processing theory.

### 3.4. Limitations and future work

The correlation between the MEG and fMRI RDMs in the MEG-fMRI fusion method amplifies the noise present in both MEG and fMRI signals, thus requiring a higher signal-to-noise ratio for MEG-fMRI fusion to attain statistical significance. Moreover, MEG-fMRI fusion only captures neural activity present in both MEG and fMRI separately. Thus, analyses along only the temporal dimension using MEG data or only the spatial dimension using fMRI data will likely yield stronger signal. Conversely, these limitations have the benefit of making the MEG-fMRI fusion technique conservative, meaning that any statistically significant results are more likely to be true effects.

All spatial information stems from fMRI and all temporal information stems from MEG, which theoretically can result in spatially and temporally ambiguous representational correlations [66]. Incorporating temporal information in the fMRI data or spatial information in the MEG data is the best way to resolve any potential ambiguity. While our slow (2 second TR) fMRI acquisition did not allow for a useful increase in temporal resolution in the fMRI data, we did increase the spatial resolution of the MEG data using source-estimation in order to minimize any potential ambiguity. Furthermore, even without incorporating temporal resolution in fMRI and spatial resolution in MEG, MEG-fMRI fusion has been shown to produce reliable spatiotemporal signals both across studies and subjects [15–22]. MEG-fMRI fusion results also demonstrate strong agreement with independently collected spatiotemporal results [15,16,67–70].

The goal of our ROI analysis was to broadly test the ventral visual cortex, abutting regions, and classical memory regions that may be implicated in visual perception. A study selecting ROIs to test specific hypotheses of an ROI’s contribution to the memorability effect can yield impactful results. Achieving source-estimated temporal information in the memory regions of hippocampus, parahippocampal gyrus, and amygdala would have allowed us to further elucidate the interplay between perception and memory, but due to the medial anatomical location of these ROIs, such MEG source-estimated information was not possible to obtain. Promising future directions include defining a temporal hierarchy for the memorability effect in light of the strong evidence of feedback processes (see section 2.2 and 2.3; [22]). Additionally, there exist many categories that might ecologically benefit from a stronger encoding into memory. We wonder if regions when sensitive to image memorability show categorical preferences, as in object recognition [71], or are agnostic to categorical information. Identifying the memorability effect’s relationship to object recognition and memory encoding and retrieval would be a dramatic step forward in understanding human visual cognition.

### 3.5. Conclusions

In this work, we elucidate the spatiotemporal dynamics of image memorability sensitivity in the brain by combing the high temporal resolution of MEG with the high spatial resolution of fMRI. We find sensitivity to image memorability in EVC, inferior temporal cortex, fusiform gyrus, lateral occipital cortex, and the banks of the STS, and we quantify onset times of sensitivity to image memorability in relation to stimulus onset. We find that High Memorable images are preferentially processed in high-level visual regions [8–10,14] and EVC late in time (but not in extra-perceptual regions). The behavioral readout of image memorability is represented after high-level feature processing in visual regions and significantly represented in classical memory regions in the MTL. In support of the levels-of-processing theory of memory, we hypothesize that High Memorable images undergo additional perceptual processing to extract detailed conceptually and socially relevant information for better encoding and retrieval from memory.

## 4. Materials and methods

### 4.1. Participants

Fifteen healthy volunteers (9 female, 27.87 ± 5.17 years old) participated in this experiment. All participants were right-handed with normal or corrected-to-normal vision and provided written consent. The studies were conducted in accordance with the Declaration of Helsinki and approved by the local Institutional Review Board ethics committee (Committee on the Use of Humans as Experimental Subjects (COUHES) of the Massachusetts Institute of Technology, approval code: 1510287948).

### 4.2. Stimuli

We selected 78 pairs of images (for a total of 156 images) from the LaMem Memorability dataset [72] and the 10K USA Adult Faces [6] to form a categorically diverse stimulus set. Each pair contained 1 High Memorable image and 1 Low Memorable image matched for high-level semantic description.

The 78 High Memorable images and 78 Low Memorable images showed no significant differences in the low-level features of color and spatial frequency (see S1 Table) [73–75]. An image’s memorability score ranges from 0 to 1 and is calculated as the decimal percentage of correct image recognition trials during a behavioral memory experiment, averaged over all participants [3]. In this study, the memorability scores are 0.854 +/− 0.089 for images in the High Memorable set and 0.519 +/− 0.105 for those in the Low Memorable set.

A memorability score magnitude RDM was computed from the stimuli’s memorability scores to reflect how each pair of images differed from one another based on human results from the memory experiment. For each pair of images *i* and *j*, the difference in magnitudes of their memorability score was entered into index (*i*,*j*) of the memorability behavior RDM. For example, 2 images *i* and *j* that were recognized equally well (i.e., the same memorability score) were represented with 0 dissimilarity at index (*i*,*j)* in the RDM. This memorability magnitude RDM was used to measure the extent that this specific behavioral readout of memorability was represented in a cortical region.

### 4.3. GIST and AlexNet stimuli features

We computed summary image statistics for each stimuli using the GIST descriptor [76,77]. These GIST features provided an additional control against image-feature confounds between the High Memorable and Low Memorable image sets not accounted for by our matching of semantics, color, and spatial frequency (see S1 Table). Specifically, the GIST features were computed by applying a series of Gabor filters across 8 orientations and 4 spatial frequencies to each image. Each image was then windowed using a 4 × 4 grid to result in a 512 × 1 vector.

We computed a High Memorable and Low Memorable GIST RDM, each of size 78 × 78, by calculating the pairwise distance (1 –Pearson correlation) between the GIST features of the 78 High Memorable and 78 Low Memorable images, respectively. We used the High and Low Memorable GIST RDMs for partial correlation analysis (for sections 2.1, 2.2, 2.3, and 2.4) as the potentially confounding variable to control for the possibility that GIST image features, not differences in memorability scores, drove the correlations seen in the results below. See S1 Fig for a visualization of the High and Low Memorable GIST RDMs.

We extracted stimuli features from the deep convolutional neural network AlexNet [46] provided by PyTorch pretrained to classify images from ImageNet [78]. Specifically, stimuli features were extracted after the first convolutional layer and after the penultimate fully connected layer, respectively, termed “features.0” and “classifiers.4” in the model definition. Separately for each layer, the features were flattened and the pairwise dissimilarity (1 –Pearson correlation) was computed between each stimulus’s feature vector and entered into an RDM. In this way, we created a 78 × 78 high memorable RDM, 78 × 78 low memorable RDM, and a 156 × 156 all conditions RDM for the AlexNet layers. The features from the first convolutional layer in AlexNet (referred to as Conv1 here) reflect low-level image features, and the features from the penultimate fully connected layer (referred to as FC7 here) reflect high-level image features useful to probe interpretable representations in the primate visual cortex [42–44].

### 4.4. Region of interest selection

We defined a total of 10 non-overlapping ROIs, 9 from the Desikan–Killiany Atlas [24] and 1 spherical ROI following Bainbridge and colleagues (2017) [8]. The Desikan–Killiany Atlas used to define the fMRI ROIs was downloaded from the AtlasReader [79] GitHub page (https://github.com/miykael/atlasreader/tree/master/atlasreader/data) in the filepath “atlasreader/atlasreader/data/atlases/atlas_desikan_killiany.nii.gz.” The atlas was created by converting FreeSurfer’s (version 6.0) [80] “aparc+aseg.mgz” file output from the FreeSurfer folder “subjects/cvs_avg35_inMNI152/mri” into a standard NifTi file using FreeSurfer’s mri_convert function. The labels follow FreeSurfer’s “FreeSurferColorLUT.txt” file. Six of the 9 Desikan–Killiany Atlas [24] ROIs spanned the ventral visual stream where we a priori might have expected to see sensitivities to image memorability [8,9,14]. Namely, we defined EVC (which was made by concatenating estimated cortical signals in Lingual, Cueneus, and Pericalcarine), lateral occipital, inferior temporal, middle temporal, fusiform, and banks of the STS [24]. We used these same ROIs for MEG source-estimation and fMRI ROI analyses. The remaining 3 ROIs from the Desikan–Killiany Atlas [24] were the classical memory regions of hippocampus, parahippocampal gyrus, and amygdala. Finally, we defined the memory region of LPFC as a spherical ROI with radius 4 voxels centered around MNI coordinate (−50, 9, 31), as in Bainbridge and colleagues (2017) [8].

### 4.5. Experimental procedure

fMRI and MEG data were acquired in separate sessions (session orders were counterbalanced) and followed presentation parameters optimized for each imaging modality. Participants passively viewed the stimuli during 1 MEG session and 2 fMRI sessions while engaged in an orthogonal vigilance task. In all sessions, images were presented at the center of the screen at 6.0° visual angle with 500 ms duration, overlaid with a black fixation cross. Subjects were not informed that images had different memorability scores.

For fMRI, participants completed 2 sessions of 5 to 8 runs of 610 s each (totaling 11 to 15 runs over both sessions). Each image was presented once per run and image order was randomized. Null trials occurred 25% of the time, where the fixation cross against a gray background was white for 100 ms before returning to its black color for the remainder of the trial. Participants were instructed to respond to the change in fixation color with a button press. Image and null trials were both 3 s (500 ms stimulus display 2.5 s fixation only).

For MEG, participants completed 1 session of 25 runs, with trials of 1 to 1.2 s each. Each image was presented once per run in a randomized order. Null trials occurred randomly every 3 to 5 trials and consisted of an image of an eye. Subjects were instructed to only blink on these null trials and respond with a button press.

### 4.6. MEG acquisition and analysis

#### 4.6.1. MEG acquisition and preprocessing

MEG signals were acquired from 306 channels (204 planar gradiometers, 102 magnetometers, Elekta Neuromag TRIUX, Elekta, Stockholm, Sweden) using a sampling rate of 1 kHz, filtered between 0.03 and 330 Hz. We applied temporal source space separation (maxfilter software, Elekta, Stockholm) [81,82] before analyzing data with Brainstorm [83]. For each trial, we extracted peri-stimulus data from −200 ms to +1,000 ms, removed baseline mean, and smoothed data with a 30 Hz low pass filter. We obtained 25 trials for each condition, session, and participant.

#### 4.6.2. MEG multivariate pattern analysis

We conducted MEG multivariate pattern analysis on each subject data independently (Fig 1C and 1G). First, we extracted preprocessed MEG signals from 200 ms before to 1,000 ms after stimulus onset and arranged the 306 sensor measurements in 306-dimensional vector patterns at each time point. This yields N pattern vectors per time point and stimulus condition. We then trained linear support vector machine (SVM) classifiers (LibSVM implementation, www.csie.ntu.edu.tw/~cjlin/libsvm) to discriminate patterns between any 2 conditions. Following previous works [14,22,84,85], we binned MEG trials randomly into groups of 3 for each condition. To reduce noise and computational complexity, we averaged the trials assigned to each bin, resulting in M pseudo trials [66,85–87]. Then, at each time point and for each pair of conditions, we assigned M-1 pseudo trials for training the SVM classifier. The trained SVM was then tested on the Mth left out vector from each of the 2 conditions. The random assignment to bins, sub-averaging, training and testing procedure were repeated for 100 times. The classifier performance (decoding accuracy), shown to be a reliable and high-SNR dissimilarity index [87], over the 100 iterations was averaged and used to populate a 156 × 156 matrix, each row and conditions indexed by the stimulus conditions. This procedure yielded one 156 × 156 symmetric matrix of decoding accuracies for every time point, referred to as MEG representational dissimilarity matrix (MEG RDM). Extracting the pairwise decoding accuracies of just the High Memorable conditions and just the Low Memorable conditions yields two 78 × 78 MEG RDMs per time point.

#### 4.6.3. MEG source-estimation

We mapped MEG signals to cortical sources using default anatomy [88] and based on Freesurfer automatic segmentation [89]. Performing multivariate pattern analysis on MEG signals in the 6 cortical regions of interest, we localized MEG representational information on that region. For source localization, using an overlapping spheres model [90], we first computed the forward model and then mapped MEG signals on the cortex using a dynamic statistical parametric mapping (dSPM) approach [91]. Pattern vectors in each of the 6 cortical regions were then extracted and employed for a multivariate pattern analysis in that region. Source-estimation was computed separately for the left and right hemispheres of each region. Once the RDM time series was computed for each hemisphere and region, the left and right hemispheres were combined.

### 4.7. fMRI acquisition and analysis

#### 4.7.1. fMRI acquisition and preprocessing

Both sessions were conducted at the Athinoula A. Martinos Imaging Center at the MIT McGovern Institute, using a 3T Siemens Trio scanner (Siemens, Erlangen, Germany) with a 32-channel phased-array head coil. In each session, we acquired structural images using a standard T1-weighted sequence (176 sagittal slices, FOV = 256 mm^2^, TR = 2530 ms, TE = 2.34 ms, flip angle = 9°). Across the 2 sessions, we acquired 11 to 15 runs of 305 volumes for each participant (gradient-echo EPI sequence: TR = 2,000 ms, TE = 29 ms, flip angle = 90°, FOV read = 200 mm, FOV phase = 100%, bandwidth 2,368 Hz/Px, gap = 20%, resolution = 3.1 mm isotropic, slices = 33, ascending acquisition).

The fMRI data were preprocessed using SPM software (Wellcome Trust Center for Neuroimaging, University College London). For each participant, fMRI data were slice-time corrected, realigned and co-registered to the T1 structural scan of the first session, and finally normalized to the standard MNI space. We fit a general linear model (GLM) to estimate the fMRI responses to the 156 images. Stimulus onsets and durations, as well as motion and run regressors were included in the GLM. All the regressors were convolved with a hemodynamic response function (canonical HRF) with time resolution of TR/N and onset of the first slice (TR = 2,000 msec, *N* = 33 slices).

#### 4.7.2. fMRI voxelwise searchlight analysis

We obtained 156 condition-specific t-maps by computing the contrast of each condition’s GLM estimate against an implicit baseline. We then computed a searchlight analysis [92,93] to calculate an RDM at every voxel for each subject (Fig 1B and 1E). Specifically, we defined a spherical searchlight of radius 4 voxels and centered it on voxel *v*. Following previous work [16,94,95], the condition-specific t-values were extracted from all voxels within the searchlight, and the dissimilarities between each condition were calculated (1-Pearson’s R) to obtain an RDM of shape (156 × 156) for voxel *v*. Each row *i* and column *j* of the RDM corresponded to a specific condition, and the *(i*,*j)* index into the RDM was the dissimilarity measure between condition *i* and condition *j*. The searchlight sphere was then moved to the next voxel, and the procedure was repeated until an RDM was calculated at each voxel. This searchlight analysis was performed on each subject separately.

#### 4.7.3. fMRI ROI searchlight analysis

To obtain 1 RDM for each of the 6 ROIs, we averaged the individual voxel RDMs (from the whole-brain searchlight procedure detailed in Methods section 4.7.2) contained within the corresponding ROI mask. We repeated this procedure for each subject separately.

#### 4.8. MEG-fMRI fusion

M/EEG-fMRI fusion uses RSA [23,41] to relate temporally resolved MEG or EEG signal with spatially resolved fMRI signal [15,17,18,96,97]. The method assumes that if 2 images are similarly represented in MEG patterns, they should also be similarly represented in fMRI patterns. Translating this neural data into representational space allows us to connect a location in the brain to a particular time point. This creates a spatiotemporally resolved view of the emergence of a specific visual representation in the brain.

#### 4.8.1. Whole-brain MEG-fMRI fusion

We used a partial correlation analysis to correlate the fMRI RDMs at each voxel (as described in section 4.7.2) with the whole-brain MEG RDMs (as described in section 4.6.2) while regressing out the GIST image feature RDMs (as described in section 4.3) (Fig 1C and 1F) in order to control for low and mid-level image features not explicitly accounted for in the stimulus set. In line with previous work [15,16], analysis was conducted on the subject-averaged MEG RDMs with the subject-specific fMRI RDMs. For each time point, we computed the similarity (Spearman’s R, thus avoiding scaling issues and parametric assumptions) between the MEG RDM and the fMRI RDM of each voxel while controlling for the potentially confounding GIST image features. This yielded a 3D correlation map of representational similarities, indicating locations in the brain at which neuronal processing emerged at a particular time point. Repeating this analysis for each time point creates a spatiotemporally resolved view of neural activity in the brain during perception free of any spatial priors.

We computed MEG-fMRI fusion separately for High Memorable and Low Memorable conditions by using their respective MEG, fMRI, and GIST High and Low Memorable RDMs. The spatiotemporal dynamics of the effect of image memorability was the difference between the High and Low Memorable MEG-fMRI fusion time series (Fig 1G). Nonparametric permutation-based cluster correction statistics (*n* = 15, cluster-definition threshold *P* < 0.001, significance threshold (alpha) *P* < 0.01, permutations = 1,000, right-tailed) were used to identify significant spatial and temporal clusters for the 4D (voxel X, voxel Y, voxel Z, time point t) correlational fusion series [98,99]. Specifically, we first computed a mean 4D correlation map by averaging each subject’s 4D correlation map. Next, we used each voxel’s values in the baseline period (−200 to 0 ms) in the mean correlation map to empirically construct a distribution of the null hypothesis. We estimated a maximal cluster size threshold by randomly shuffling the signs of each subject’s data 1,000 times, averaging over the subjects, and defining clusters by the values’ spatial and temporal proximity at the cluster-definition threshold (defined to be right-sided, *P* = 0.001). An empirical distribution of the maximal cluster size was computed via permutation sampling and a cluster size threshold of *P* = 0.01. A cluster is significant if it exceeds the cluster size threshold.

#### 4.8.2. Source-estimated MEG-fMRI ROI fusion

ROI-based MEG-fMRI fusion, as used in this study, first required the calculation of source-estimated MEG-fMRI fusion. We first computed a given ROI’s source-estimated MEG RDM time series (as described in section 4.6.3). We then computed a given ROI’s fMRI RDM by averaging each voxel’s RDM contained within the ROI mask (as described in section 4.7.3). Finally, we performed a partial correlation between the subject-averaged MEG ROI RDMs at each time point and the subject-specific fMRI ROI RDM with the GIST RDM serving as the potentially confounding variable. This procedure resulted in a correlation time series for each ROI of size 15 × 1,201 (n_subjects × n_timepoints).

When determining the onset latencies of the ROI fusion correlation time series, we faced 2 main challenges: low signal-to-noise ratio (as common with most neuroimaging data) and no prior knowledge of the signal’s canonical shape. We addressed these 2 challenges using a jackknife-based baseline criteria technique. Starting with an ROI correlation time series matrix of size 15 × 1,201 (n_subjects × n_timepoints), we followed previous work to identify significant latencies [37–40].

First, we computed the standard deviation of the baseline values across time (−200 to 0 ms) from the 15-subject grand-averaged correlation time series to establish the baseline criteria. Next, we iterated over each subject to compute 15 subsamples of leave-one-out mini grand averages, where each of the remaining 14 subjects are averaged at each iteration (jackknife) [38]. For each of the jackknifed subsamples, a time point t was considered significant if it satisfied 2 criteria [37,39]: (1) the value at time point t was greater than or equal to 2 times the baseline standard deviation; and (2) the average value of the next 10 windows of t + 50 ms was also greater than or equal to 2 times the baseline standard deviation. The first significant time point for each of the 15 jackknifed subsamples was the onset for that subsample. This procedure resulted in 15 samples of onset latencies per condition.

The strict baseline criteria ensured that significant time points satisfy an amplitude criterion (2 times the baseline standard deviation) and a temporal grouping criterion (10 moving windows of 50 ms) that noise was unlikely to satisfy, and the jackknifing procedure increased the signal-to-noise ratio to better estimate signals with non-canonical shapes. For an ROI’s High Memorable, Low Memorable, or High > Low Memorable curves to be considered significant, at least 1 time point must exist where all 15 subsamples passed the 2 criteria. Otherwise, the correlation curve was considered not significant.

Since the subsamples were no longer independent, we adjusted the variance by multiplying by a factor of (*n*—1) [2] (where *n* = 15) when computing subsequent statistics (for detailed proof and discussion of this adjustment, see [37,38,100]). The mean onset latency of each condition and ROI was computed by averaging the onset latencies of the 15 subsamples. The 95% confidence interval around the onset latency for each condition and ROI was computed by multiplying the standard error of the onset latencies (with the adjusted variance) by a critical t-statistic of 2.145 to represent a 95% confidence interval with 14 degrees of freedom. Onset latencies were computed with a one-sided 1 sample *t* test (against an onset of 0 ms, stimulus onset), and onset latency differences between conditions (as in section 2.3) were computed with a two-sided paired *t* test.

Statistical tests for latency differences between conditions (section 2.3) and memorability magnitude to memory RDM correlations (section 2.4) were corrected for multiple comparisons using Bonferroni–Holm correction (*n* = 12 tests and *n* = 4 tests, respectively) at q = 0.05 [101].

## Supporting information

S1 Fig Visualization of RDMs. We show the RDMs for the 9 fMRI ROIs, memorability score magnitudes, and GIST features for the 78 High Memorable image conditions, 78 Low Memorable image conditions, all 156 image conditions. The fMRI and GIST RDMs are computed by calculating the pairwise dissimilarity (1—Pearson correlation) between each stimulus’s feature vector. The memorability magnitude RDM is computed by calculating the magnitude of difference in memorability scores between each pair of images. For the GIST RDMs, each stimulus feature is a 512 × 1 vector computed from a GIST model. For the fMRI RDMs, each stimulus feature is a n_voxel × 1 vector of t-values, where n_voxels is the number of voxels contained within a spherical searchlight (radius of 4 voxels). All RDMs contained within the ROI’s mask are averaged together, and the average RDM over 15 subjects is shown here. The dissimilarity is ranked and divided by the maximum dissimilarity, resulting in the displayed RDMs with percent values.
(TIF)

S2 Fig fMRI-only univariate memorability contrast. For each subject, a GLM was used to fit the observed fMRI data to the experimental conditions. The beta weight map from all 78 high memorable conditions was contrasted against the beta weight map from all 78 low memorable conditions to create a high memorable > low memorable contrast image (one-sample *T* test, *p* < 0.001, voxel extent threshold (k) = 10). Each high memorable > low memorable contrast image was input into a group level analysis to compute the group effect (one-sample *T* test, *p* < 0.001, voxel extent threshold (k) = 10), shown here. The colorbar scale has units of t-statistic.
(TIF)

S3 Fig fMRI-only multivariate memorability contrast. For each subject, a GLM was used to fit the observed fMRI data to the experimental conditions. The beta weight map from each of the 156 conditions was contrasted against the beta weight map from response (null) trials to create a t-statistic significance map (referred to as t-map) for each condition (one-sample *T* test, *p* < 0.001, voxel extent threshold (k) = 10). A searchlight analysis was then performed on these t-maps to create a 156 × 156 RDM at each voxel in the brain, where each (i, j) index into the RDM is a measure of the dissimilarity between condition i and condition j. We then created a single hypothesized 156 × 156 RDM where each (i, j) index was the Euclidean distance (absolute value of the difference) between the memorability score of condition i and the memorability score of condition j. The memorability score associated with each condition is a high-level behavioral measure of image memorability, and therefore, the hypothesized RDM is a high-level behavioral measure of image memorability in representation space. We then correlated (Spearman’s R) the hypothesized RDM with each RDM at each voxel in the brain and perform statistical analysis on the correlation map shown here (cluster statistics, *n* = 15, cluster-definition threshold *P* < 0.01, cluster threshold *P* < 0.01, permutations = 1,000).
(TIF)

S1 Table High and Low Memorable stimuli controlled for low-level image features. We use the Natural Image Statistical Toolbox [73,74,101] to compare low-level statistics (color, brightness, contrast, and spatial frequency at various energy levels) across the set of 78 High Memorable images and the set of 78 Low Memorable images. The spectral energy levels of 10%, 30%, 50%, 70%, and 90% refer to the percentage of total energy of the spectrum in the image sets (larger percentages encompass the energy levels of lesser percentages). In RGB color space, the “R,” “G,” and “B” denote significance testing within the “Red,” “Green,” and “Blue” color channels, respectively. In Lab color space, “L,” “a,” and “b” denote significance testing within “Luminance,” “a color channel,” and “b color channel,” respectively. At a *p*-value of *p* < 0.05, no statistically significant differences were found.
(XLSX)

S2 Table Source-estimated ROI-based MEG-fMRI fusion onset latency. The mean latency and 95% one-sided confidence intervals (milliseconds) for the source-estimated MEG-fMRI ROI fusion correlation time series in the High Memorable, Low Memorable, High > Low Difference, and High > Low Difference minus High Memorable conditions were computed using a jackknife-based relative baseline criteria on the searchlight ROI-based MEG-fMRI fusion correlation time series. For each condition, the standard deviation of the 15 subject grand-averaged baseline values was first computed (from −200–0 ms before stimulus onset). Then, for each of the jackknife subsamples, any time point that met the (1) amplitude criteria of a value greater than or equal to the baseline value and (2) the temporal criteria of an averaged value in 10 consecutive 50 ms windows greater than or equal to the baseline value was deemed significant. The first significant time point in each of the jackknife subsamples (*n* = 15) was used to compute the average latency and 95% confidence interval. The variance in the standard error calculation was corrected by a factor of (*n*– 1) [2] to account for the dependence between the jackknifed subsamples. A time series that contained at least 1 subsample with zero significant time points (that did not meet the 2 criteria stated above) was deemed “Not Significant,” denoted in the table by “N.S.”
(XLSX)

S3 Table Model MEG RSA latency. The mean latency and 95% one-sided confidence intervals (milleseconds) for the source-estimated MEG-model RDM correlation time series for AlexNet Conv1, AlexNet FC7, and memorability magnitude model RDMs were computed using a jackknife-based relative baseline criteria. For each correlation time series, the standard deviation of the 15 subject grand-averaged baseline values was first computed (from −200–0 ms before stimulus onset). Then, for each of the jackknife subsamples, any time point that met the (1) amplitude criteria of a value greater than or equal to the baseline value and (2) the temporal criteria of an averaged value in 10 consecutive 50 ms windows greater than or equal to the baseline value was deemed significant. The first significant time point in each of the jackknife subsamples (*n* = 15) was used to compute the average latency and 95% confidence interval (one-sided *t* test). For the latency difference calculations between MEG-model correlation time series, a two-sided paired *t* test was used. The variance in the standard error calculation was corrected by a factor of (*n*– 1) [2] to account for the dependence between the jackknifed subsamples. In the latency difference calculations, multiple comparisons were corrected (*n* = 12 comparisons) using Bonferroni–Holm correction [100] at q = 0.05. The *p*-values shown here are adjusted for Bonferroni–Holm multiple comparison correction, but the 95% confidence intervals are not adjusted to retain transparency of the original computation and avoid misinterpretations from the step-wise corrections employed by the Bonferroni–Holm method.
(XLSX)

S4 Table Parametric and nonparametric statistics for memory ROI correlations with memorability magnitude RDM. For each of the 4 memory regions studied here, the correlation between the fMRI ROI 156 × 156 RDM and the 156 × 156 memorability magnitude RDM was computed (Spearman, partialled out GIST features). Significance was computed using a one-sided *t* test (parametric) and one-sided Wilcoxon sign rank test (nonparametric). Both tests were corrected for multiple comparisons (*n* = 4 comparisons, Bonferroni–Holm correction) at q = 0.05 [100]. The adjusted *p*-value is shown.
(XLSX)

S1 Movie Whole-brain MEG-fMRI fusion movies. Youtube link: https://youtu.be/skYb5l5xWBQ. The video depicts axial slices of the whole-brain MEG-fMRI fusion movies for the High Memorable images, Low Memorable images, and the difference between the High and Low Memorable fusion correlation time series (High > Low Memorable difference). The 3 movies were computed independently with identical statistics (permutation-based cluster statistics; cluster-definition threshold = 0.001, significance threshold (alpha) = 0.01, n_permutations = 1,000) and stitched together in the one movie linked here for easy side-by-side comparison.
(MP4)

## Abbreviations

dSPM: dynamic statistical parametric mapping
EVC: early visual cortex
fMRI: functional magnetic resonance imaging
GLM: general linear model
LPFC: left prefrontal cortex
MEG: magnetoencephalography
MTL: medial temporal lobe
RDM: representational dissimilarity matrix
ROI: region of interest
RSA: representational similarity analysis
STS: superior temporal sulcus
SVM: support vector machine
