# Supplementary figures and images for: Visual perception of highly memorable images is mediated by a distributed network of ventral visual regions that enable a late memorability response
Source: PLoS Biol. 2024 Apr 1;22(4):e3002564. doi: 10.1371/journal.pbio.3002564 (PMC10984539; doi:10.1371/journal.pbio.3002564)

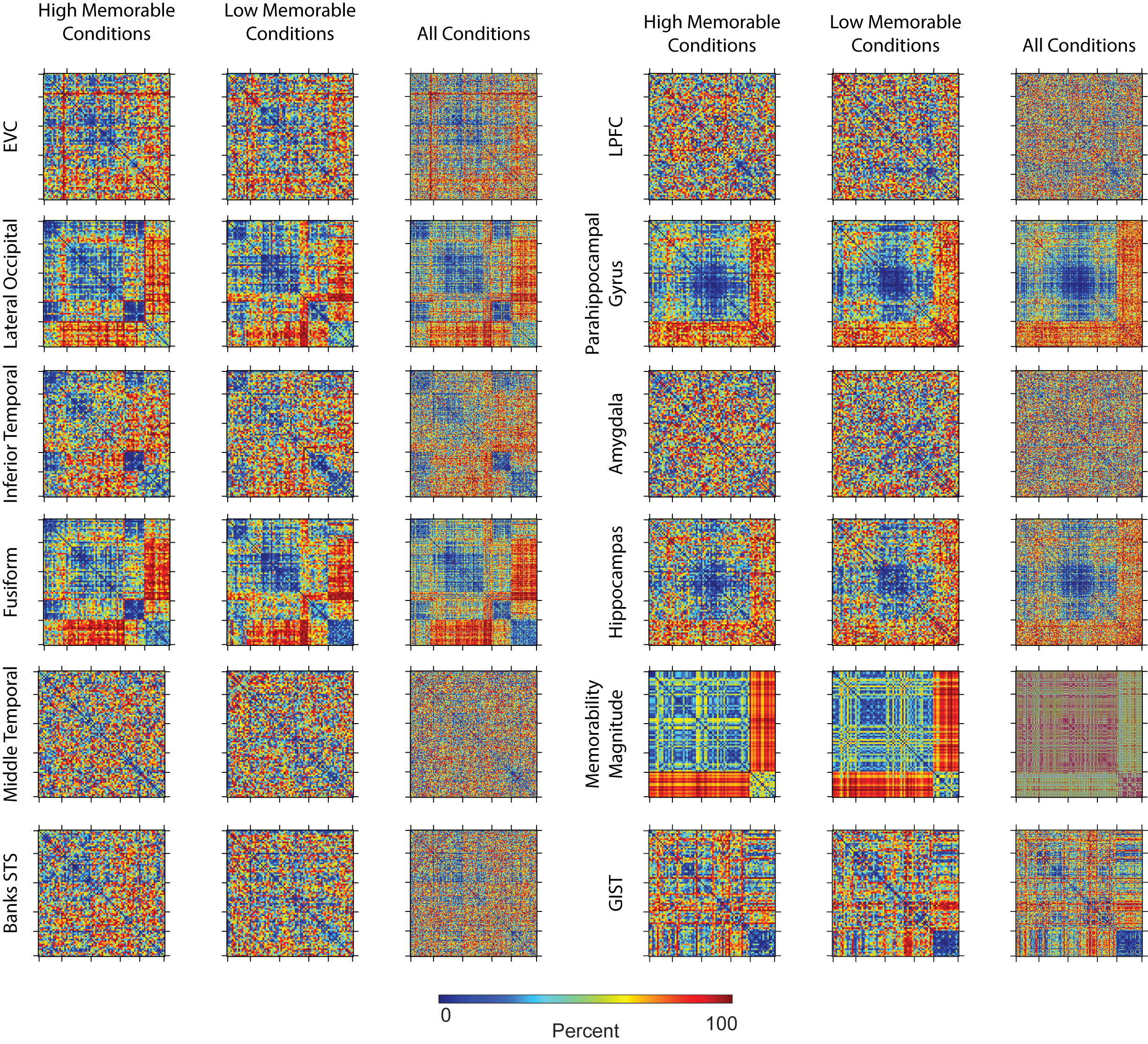

Supplement: S1 Fig — We show the RDMs for the 9 fMRI ROIs, memorability score magnitudes, and GIST features for the 78 High Memorable image conditions, 78 Low Memorable image conditions, all 156 image conditions. The fMRI and GIST RDMs are computed by calculating the pairwise dissimilarity (1—Pearson correlation) between each stimulus’s feature vector. The memorability magnitude RDM is computed by calculating the magnitude of difference in memorability scores between each pair of images. For the GIST RDMs, each stimulus feature is a 512 × 1 vector computed from a GIST model. For the fMRI RDMs, each stimulus feature is a n_voxel × 1 vector of t-values, where n_voxels is the number of voxels contained within a spherical searchlight (radius of 4 voxels). All RDMs contained within the ROI’s mask are averaged together, and the average RDM over 15 subjects is shown here. The dissimilarity is ranked and divided by the maximum dissimilarity, resulting in the displayed RDMs with percent values. (TIF) [file pbio.3002564.s001.tif]

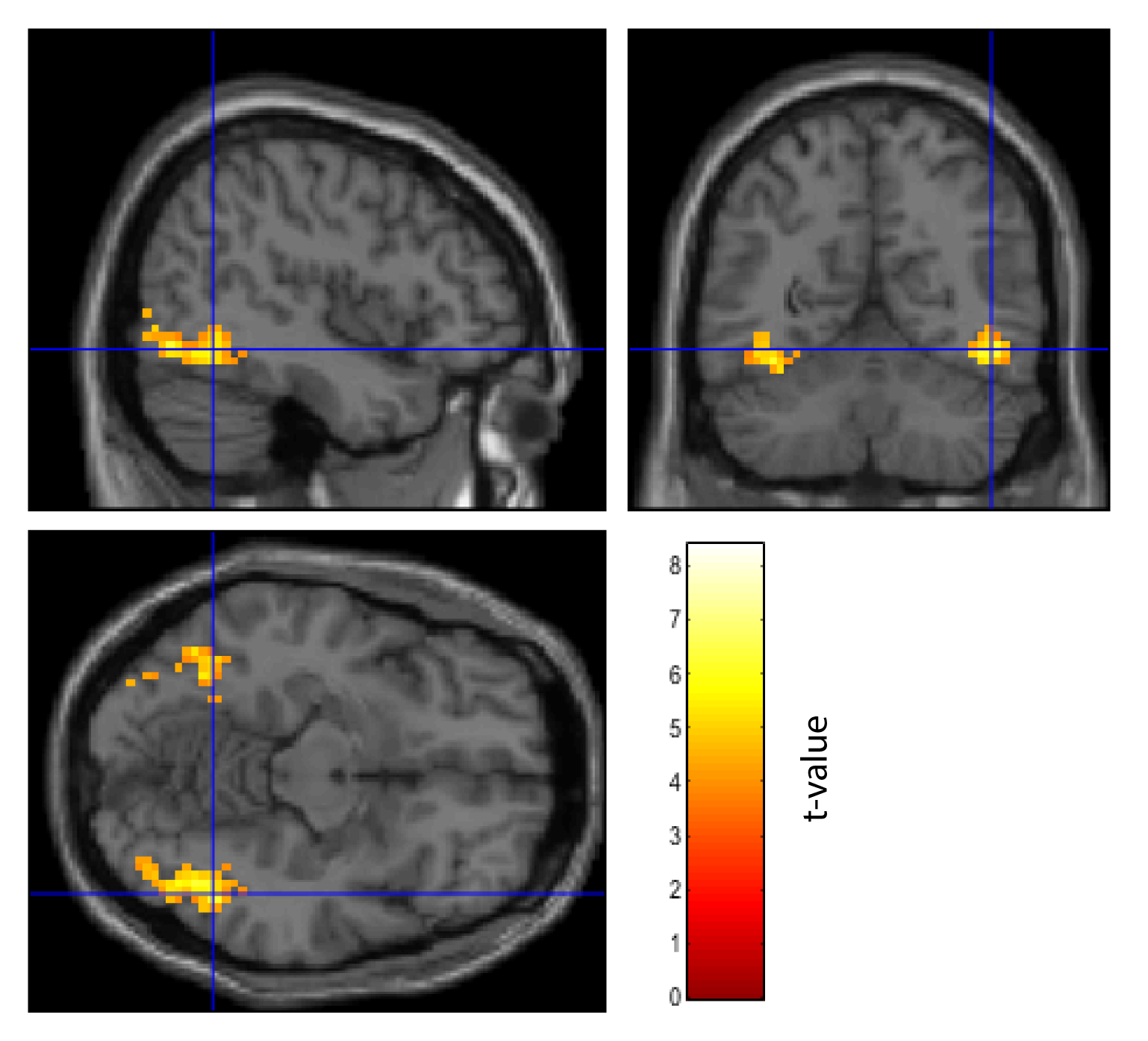

Supplement: S2 Fig — For each subject, a GLM was used to fit the observed fMRI data to the experimental conditions. The beta weight map from all 78 high memorable conditions was contrasted against the beta weight map from all 78 low memorable conditions to create a high memorable > low memorable contrast image (one-sample T test, p < 0.001, voxel extent threshold (k) = 10). Each high memorable > low memorable contrast image was input into a group level analysis to compute the group effect (one-sample T test, p < 0.001, voxel extent threshold (k) = 10), shown here. The colorbar scale has units of t-statistic. (TIF) [file pbio.3002564.s002.tif]

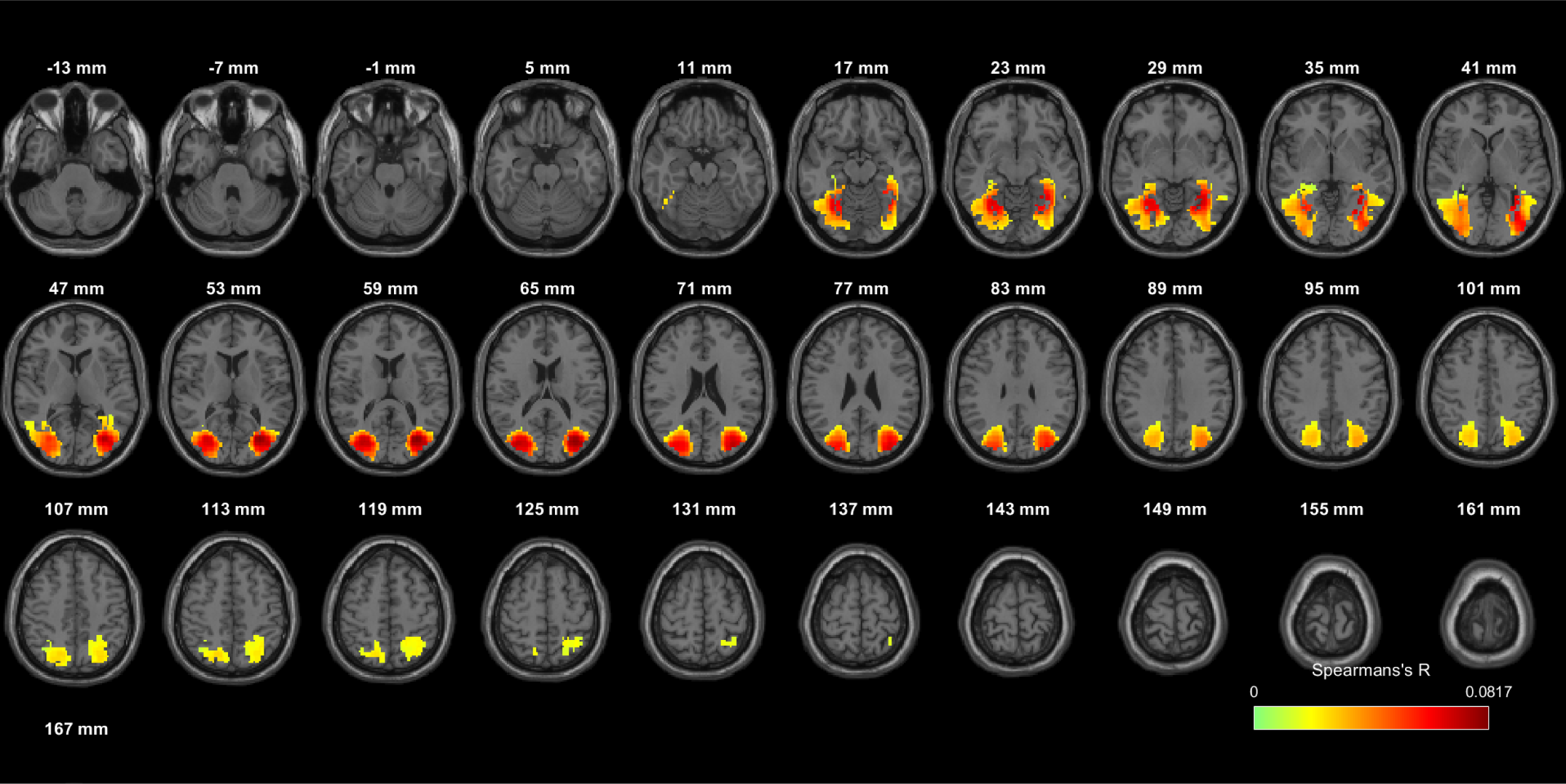

Supplement: S3 Fig — For each subject, a GLM was used to fit the observed fMRI data to the experimental conditions. The beta weight map from each of the 156 conditions was contrasted against the beta weight map from response (null) trials to create a t-statistic significance map (referred to as t-map) for each condition (one-sample T test, p < 0.001, voxel extent threshold (k) = 10). A searchlight analysis was then performed on these t-maps to create a 156 × 156 RDM at each voxel in the brain, where each (i, j) index into the RDM is a measure of the dissimilarity between condition i and condition j. We then created a single hypothesized 156 × 156 RDM where each (i, j) index was the Euclidean distance (absolute value of the difference) between the memorability score of condition i and the memorability score of condition j. The memorability score associated with each condition is a high-level behavioral measure of image memorability, and therefore, the hypothesized RDM is a high-level behavioral measure of image memorability in representation space. We then correlated (Spearman’s R) the hypothesized RDM with each RDM at each voxel in the brain and perform statistical analysis on the correlation map shown here (cluster statistics, n = 15, cluster-definition threshold P < 0.01, cluster threshold P < 0.01, permutations = 1,000). (TIF) [file pbio.3002564.s003.tif]
